# FOXO3-engineered human mesenchymal progenitor cells efficiently promote cardiac repair after myocardial infarction

**DOI:** 10.1007/s13238-020-00779-7

**Published:** 2020-08-18

**Authors:** Jinghui Lei, Si Wang, Wang Kang, Qun Chu, Zunpeng Liu, Liang Sun, Yun Ji, Concepcion Rodriguez Esteban, Yan Yao, Juan Carlos Izpisua Belmonte, Piu Chan, Guang-Hui Liu, Weiqi Zhang, Moshi Song, Jing Qu

**Affiliations:** 1 Advanced Innovation Center for Human Brain Protection, National Clinical Research Center for Geriatric Disorders, Xuanwu Hospital Capital Medical University, Beijing, 100053 China; 2 State Key Laboratory of Membrane Biology, Institute of Zoology, Chinese Academy of Sciences, Beijing, 100101 China; 3 State Key Laboratory of Stem Cell and Reproductive Biology, Institute of Zoology, Chinese Academy of Sciences, Beijing, 100101 China; 4 Disease Genomics and Individualized Medicine Laboratory, Beijing Institute of Genomics, Chinese Academy of Sciences, Beijing, 100101 China; 5 Institute of Stem Cell and Regeneration, Chinese Academy of Sciences, Beijing, 100101 China; 6 University of Chinese Academy of Sciences, Beijing, 100049 China; 7 The MOH Key Laboratory of Geriatrics, Beijing Hospital, National Center of Gerontology, Beijing, 100730 China; 8 Department of Cardiology, Beijing Anzhen Hospital, Capital Medical University, Beijing, 100029 China; 9 Gene Expression Laboratory, Salk Institute for Biological Studies, La Jolla, 92037 USA

**Dear Editor,**

Myocardial infarction (MI) is the irreversible cardiomyocyte death resulting from prolonged oxygen deprivation due to obstructed blood supply (ischemia), leading to contractile dysfunction and cardiac remodeling. In recent decades, stem cell transplantation has been extensively investigated for the repair of injured heart in animal studies and clinical trials (Kanelidis et al., 2017; Gyongyosi et al., 2018). Among cell-based therapies in clinical development, mesenchymal progenitor cells (MPCs) are attractive candidates due to their multi-lineage potential and immunomodulatory properties (Bagno et al., 2018). However, low quality (e.g., reduced proliferative ability and increased cellular senescence at late passages) and heterogeneous cell sources, as well as poor retention and survival rate of transplanted MPCs in an *in vivo* niche present obstacles towards broader clinical applications (Nguyen et al., 2016; Li et al., 2020).

*Forkhead box O3* (*FOXO3*), one of the most prominent genes related to human longevity, functions in diverse biological processes including DNA repair, oxidative stress response, cell proliferation and cellular senescence (Liu et al., 2018). We have previously reported that FOXO3 loss drives primate arterial aging and that constitutive activation of FOXO3 in human embryonic stem cell (hESC)-derived MPCs enhances their stress resistance and attenuates cellular senescence (Yan et al., 2019; Zhang et al., 2020). Here, we evaluated the cardiac repair after MI in immunodeficient mice intramyocardially transplanted with FOXO3-genetically-enhanced MPCs (FOXO3-GE-MPCs).

FOXO3-GE-MPCs were generated by directed differentiation of hESCs in which two FOXO3 phosphorylation sites were replaced with alanine (S253A, S315A) using targeted gene editing (Fig. S1A and S1B). The engineered FOXO3 could not be phosphorylated by AKT at S253 or S315 and was therefore constitutively active in the nucleus (Yan et al., 2019). Consistent with previous observations (Yan et al., 2019), FOXO3-GE-MPCs exhibited increased proliferation and decreased senescence-associated (SA)-β-gal activity relative to wildtype MPCs (WT-MPCs) (Fig. S1C–E). We next investigated whether FOXO3-GE-MPCs would be retained longer in the heart than WT-MPCs when intramyocardially delivered at the initiation of myocardial ischemia. *In vivo* imaging of luciferase-labelled MPCs revealed that transplanted WT-MPCs diminished within five days whereas FOXO3-GE-MPCs remained detectable until day 11 (Fig. 1A). Due to the limited resolution and sensitivity of *in vivo* imaging, we performed immunofluorescence staining of the human Golgi marker hTGN46 and RT-PCR of human *GAPDH* to further detect the transplanted cells in ischemic hearts and found that FOXO3 enhancement prolonged MPC retention up to 4 weeks after MI (Fig. 1B and 1C).

**Figure 1:**
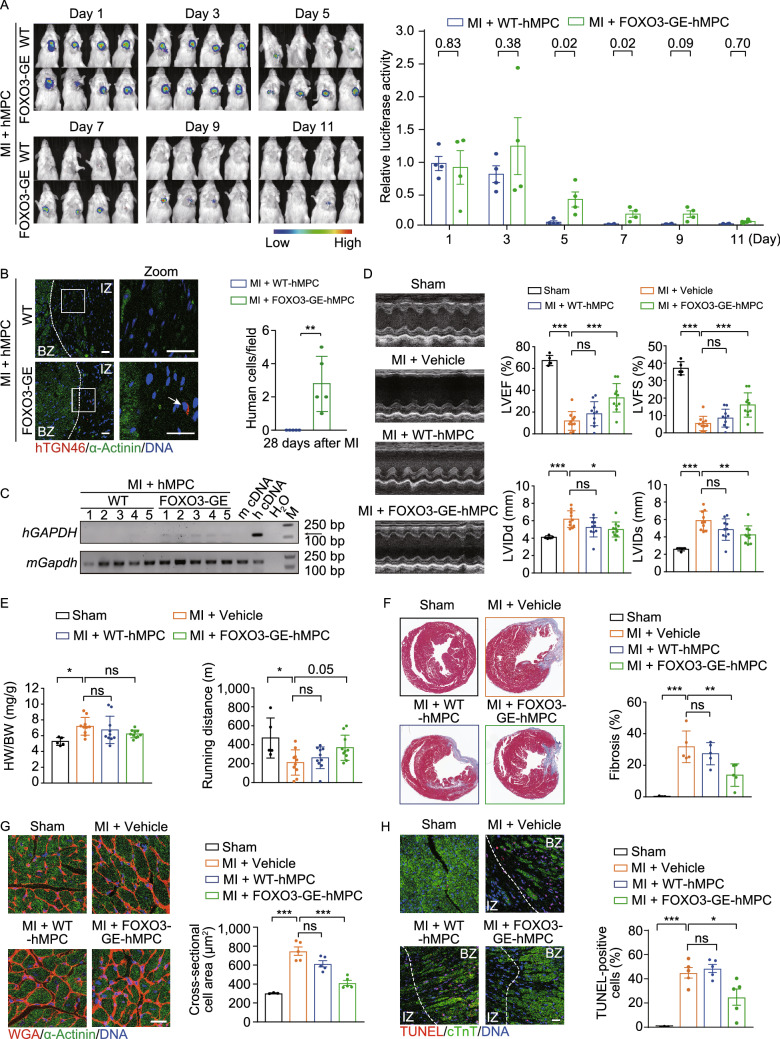
**FOXO3-GE-MPCs ameliorate cardiac dysfunction and left ventricular remodeling after myocardial infarction**. (A) hMPC retention evaluation using *in vivo* tracing of luciferase-labelled MPCs along with quantitative analysis. NOD-SCID mice with left anterior descending coronary artery ligation (LAD) were subjected to intramyocardial injection of luciferase-labelled WT-MPCs or FOXO3-GE-MPCs. Luciferase activity was detected and quantified using an *in vivo* imaging system (IVIS) at days 1, 3, 5, 7, 9 and 11 after the transplantation. Quantitative data of luciferase activity are presented as the mean ± SEMs. *n* = 4. *P* values for each time point are as indicated (two-tailed *t* test). (B) Representative immunostaining images of the human Golgi marker hTGN46 (red) and the mouse myocardial marker α-actinin (green) in mouse heart sections. Areas in white rectangles are enlarged to the right. Scale bar, 25 μm. The white dotted line denotes the lesion border of the heart. BZ, border zone; IZ, infarct zone. Quantitative data showing the numbers of resident donor cells at 4 weeks after the transplantation are presented as the mean ± SEMs. *n* = 5. **, *P* < 0.01 (two-tailed *t* test). (C) RT-PCR analysis of human GAPDH (*hGAPDH*) mRNA expression in the infarct border zone. Mouse GAPDH (*mGapdh*) was used as an internal control. Black arrow points to the expected band of hGAPDH amplicons. (D) Representative echocardiographic images at 4 weeks after myocardial infarction (MI) and intramyocardial injection of vehicle, WT-MPCs or FOXO3-GE-MPCs. Quantitative data on left ventricular ejection fraction (LVEF), left ventricular fractional shortening (LVFS), left ventricular internal diameter in diastole (LVIDd) and left ventricular internal diameter in systole (LVIDs) are presented as the mean ± SEMs to the right. *n* = 5 for sham group; *n* = 10 for the other groups. *, *P* < 0.05; **, *P* < 0.01; ***, *P* < 0.001; ns, not significant (one-way ANOVA followed by Dunnett’s test). (E) Ratio of heart weight to body weight (HW/BW) and running distance during exercise exhaustion test. Data are shown as the mean ± SEMs. *n* = 5 for sham group; *n* = 10 for the other groups. *, *P* < 0.05; ns, not significant (one-way ANOVA followed by Dunnett’s test). (F) Masson’s trichrome staining. Quantitative data are shown as the mean ± SEMs. *n* = 3 for sham group; *n* = 5 for the other groups. **, *P* < 0.01; ***, *P* < 0.001; ns, not significant (one-way ANOVA followed by Dunnett’s test). (G) Measurement of cardiomyocyte cross-sectional areas in mouse hearts co-stained with WGA (red) and anti-α-actinin antibody (green). Scale bar, 25 μm. Quantitative data are shown as the mean ± SEMs. *n* = 3 for sham group; *n* = 5 for the other groups. ***, *P* < 0.001; ns, not significant (one-way ANOVA followed by Dunnett’s test). (H) Evaluation of apoptotic cardiomyocytes in mouse hearts co-stained with TUNEL staining (red) and cardiac troponin T antibody (green). Scale bar, 25 μm. The white dotted line denotes the lesion border of the heart. BZ, border zone; IZ, infarct zone. Quantitative data are shown as the mean ± SEMs. *n* = 3 for sham group; *n* = 5 for the other groups. *, *P* < 0.05; ***, *P* < 0.001; ns, not significant (one-way ANOVA followed by Dunnett’s test)

Next, we used transthoracic echocardiography to explore whether FOXO3-GE-MPCs could ameliorate cardiac dysfunction and left ventricular (LV) remodeling after MI. In control mice (MI + vehicle group), we observed decreased cardiac contractility along with enlarged LV chamber at 4 weeks after MI. These effects were partially reversed by the transplantation with FOXO3-GE-MPCs, but not WT-MPCs (Fig. 1D). Similarly, an MI-induced increase in heart to body weight ratio and a decrease in running distance (Fig. 1E), along with cardiac fibrosis, compensatory hypertrophy and cardiomyocyte apoptosis, were all ameliorated only by FOXO3-GE-MPC transplantation (Figs. 1F–H and S1F). Collectively, our data indicate that FOXO3 enhancement promotes cardiac repair by MPCs after MI, suggesting that FOXO3-GE-MPCs may provide effective biomaterials for stem cell-based therapy against ischemic heart diseases.

To dissect the underlying mechanisms of cardiac repair by FOXO3-GE-MPCs, we performed RNA-seq analysis of heart tissues of the infarct border zone (Figs. 2A, 2B and S1G–J) and identified a panel of MI-upregulated genes that were reversed by the transplantation of FOXO3-GE-MPCs, including those involved in inflammatory response (Fig. 2B). In addition, RelA (p65) that is a subunit of the NF-κB transcription complex (Wang et al., 2018) was upregulated in ischemic hearts and its upregulation was attenuated only by the transplantation with FOXO3-GE-MPCs (Figs. 2C and S1K). Some of the MI-upregulated genes rescued upon FOXO3-GE-MPC transplantation were enriched in NF-κB pathway (Fig. S1L). Furthermore, several NF-κB target genes including *Cxcl13*, *Mmp9*, *Itgam*, *Tlr2* and *Hmox1* were upregulated after MI and attenuated by FOXO3-GE-MPC delivery as verified by RT-qPCR (Fig. S2A). Likewise, ORF1p, which is encoded by the autonomous non-LTR retrotransposon LINE-1 and involved in the induction of IFN-I and other pro-inflammatory cytokines (De Cecco et al., 2019), was increased upon MI and decreased by the transplantation of FOXO3-GE-MPCs (Figs. 2C and S2B). The serum levels of pro-inflammatory factors including TNF-α, IL-1β and IFN-γ were also increased after MI and reversed only by FOXO3-GE-MPCs (Fig. 2D). Altogether, these findings suggest that the transplantation of FOXO3-GE-MPCs attenuates inflammatory response after MI, which may partially explain the cardioprotective effects of FOXO3-GE-MPCs.

**Figure 2:**
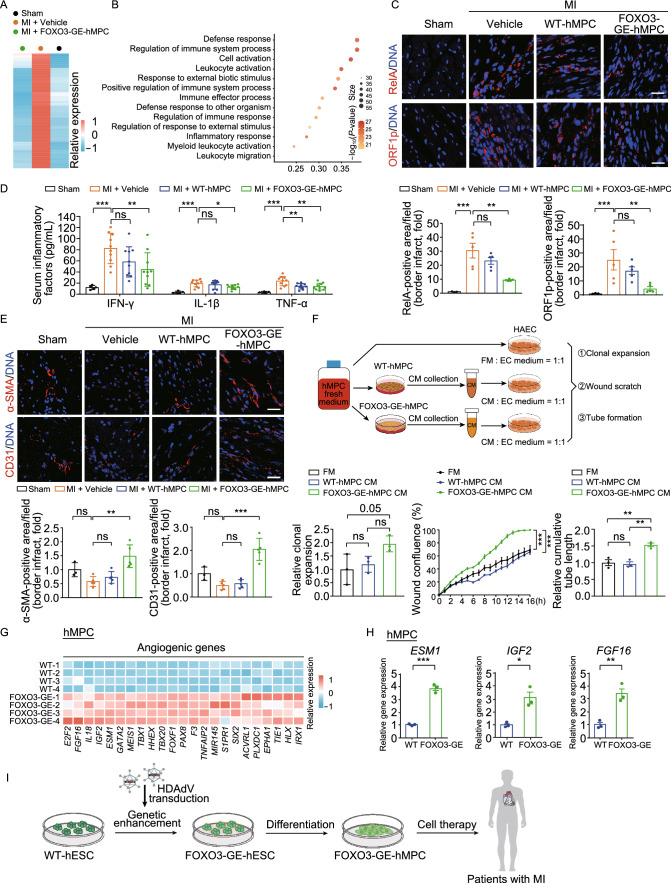
**FOXO3-GE-MPCs suppress inflammatory response and promote angiogenesis after myocardial infarction**. (A) Heatmap showing the relative expression levels of genes upregulated after MI and downregulated upon FOXO3-GE-MPC transplantation post MI. (B) Gene ontology (GO) analysis of genes in panel (A). The size of each circle represents the number of genes for each term. The value of the abscissa represents the ratio of gene number for each term to total gene number. (C) Representative immunofluorescence images and quantitative data of RelA-positive cells and ORF1p-positive cells in the infarct border zone at 4 weeks after MI, Scale bar, 25 μm. *n* = 3 for sham group; *n* = 5 for the other groups, **, *P* < 0.01; ***, *P* < 0.001; ns, not significant (one-way ANOVA followed by Dunnett’s test). (D) Serum levels of inflammatory factors (IFN-γ, IL1-β and TNF-α) measured by immunoradiometric assay. *n* = 5 for sham group; *n* = 10 for the other groups. Data are shown as the mean ± SEMs. *, *P* < 0.05; **, *P* < 0.01; ***, *P* < 0.001; ns, not significant (one-way ANOVA followed by Dunnett’s test). (E) Representative immunofluorescence images of the vascular smooth muscle cell marker α-SMA and the endothelial cell marker CD31 in the infarct border zone at 4 weeks post MI. Quantitative data at the bottom are shown as the mean ± SEMs. *n* = 3 for sham group; *n* = 5 for the other groups. Scale bar, 25 μm. **, *P* < 0.01; ***, *P* < 0.001; ns, not significant (one-way ANOVA followed by Dunnett’s test). (F) *In vitro* examination of paracrine angiogenic effects of FOXO3-GE-MPCs on HAECs. Upper, experimental schematic of the preparation of conditioned medium, cell culture and assay design. Lower, analyses of relative clonal expansion ability (left), migration ability (middle) and relative tube formation capacity (right) of HAECs after cultured with indicated media. FM, fresh medium; CM, conditioned medium. Data are shown as the mean ± SEMs. *n* = 3. **, *P* < 0.01; ***, *P* < 0.001; ns, not significant. One-way ANOVA followed by Dunnett’s test was used for clonal expansion and tube formation assays. Two-way ANOVA followed by Dunnett’s test was used for wound scratch assay. (G) Heatmap showing the relative expression levels of upregulated angiogenic genes in FOXO3-GE-MPCs as compared to WT-MPCs (P7) by RNA-seq analysis. DEGs were identified by a threshold of |log_2_ (fold change)| > 1 and adjusted *P* value (padj) < 0.05. (H) RT-qPCR analysis of the relative expression levels of secretory angiogenesis-related factors *ESM1*, *IGF2* and *FGF16* in FOXO3-GE-MPCs compared to those in WT-MPCs. Data are shown as the mean ± SEMs. *n* = 3. *, *P* < 0.05; **, *P* < 0.01; ***, *P* < 0.001 (two-tailed *t* test). (I) Schematic illustration showing the potential clinical applications of FOXO3-GE-hMPCs for the treatment of myocardial infraction. FOXO3-GE-hESCs are generated by gene editing using a helper-dependent adenovirus vector (HDAdV) and differentiated into FOXO3-GE-hMPCs for the treatment of myocardial infarction. With higher *in vivo* cardioreparative capacity, FOXO3-GE-hMPCs may serve as a better option for stem cell-based therapies against ischemic heart diseases

Given that neovascularization is essential for the repair of heart damage, we investigated whether it might underlie the cardioprotective effects of FOXO3-GE-MPCs in ischemic hearts. Indeed, we found more α-SMA-positive cells and CD31-positive cells in the infarct border zone of mouse hearts transplanted with FOXO3-GE-MPCs at 4 weeks after MI as compared to those treated with vehicle or WT-MPCs (Figs. 2E, S2C and S2D), suggesting that neovascularization may occur in the infarct border zone upon the transplantation with FOXO3-GE-MPCs and facilitate cardiac repair. To further dissect how neovascularization could be induced by the transplanted FOXO3-GE-MPCs, we evaluated the possible paracrine effects of FOXO3-GE-MPCs by culturing human aortic endothelial cells (HAECs) in different media. Improved migration and tube formation capacities along with an increasing trend in proliferative ability were observed in HAECs cultured in conditioned medium harvested from FOXO3-GE-MPCs relative to HAECs cultured in fresh MPC medium or conditioned medium harvested from WT-MPCs (Figs. 2F and S2E–G). Consistently, RNA-seq analysis of FOXO3-GE-MPCs revealed the upregulation of a list of angiogenic genes as compared to those in their wildtype counterparts, including secretory factors *ESM1*, *IGF2* and *FGF16* that were verified by RT-qPCR (Fig. 2G and 2H). Altogether, these results suggest that FOXO3-GE-MPCs promote cardiac repair after MI at least partially in a paracrine angiogenic manner.

In this study, we demonstrated that intramyocardial transplantation of FOXO3-GE-MPCs exhibited enhanced cardioprotective effects than WT-MPCs did against myocardial infarction in mice. MPCs have been used in preclinical and clinical settings against ischemic cardiac diseases (Kanelidis et al., 2017; Bagno et al., 2018; Gyongyosi et al., 2018). However, primary MPCs isolated from various human tissues, including bone marrow, adipose tissue and umbilical cord, possess diverse characteristics that aggravate cellular heterogeneity, likely resulting in batch-dependent effects (Le Blanc and Davies, 2018). The age of the donor may also affect the quality of primary MPCs as it has been reported that MPCs from older donors proliferate slowly and partially lose their stem cell characteristics (Block et al., 2017). In addition, upon serial passaging, primary MPCs exhibit the onset of senescence phenotypes and progressive loss of self-renewal and differentiation abilities, further compromising the quality and quantity of MPCs for cell therapy. By comparison, directed differentiation from pluripotent stem cells has emerged as a new strategy to generate a large number of high-quality MPCs, providing unprecedented and valuable cell resources for preclinical and clinical applications. Here, our study indicates that genetic activation of FOXO3 represents a novel strategy to generate even superior and safer cell materials for MPC-based therapies against ischemic cardiac diseases (Fig. 2I).

A major challenge of MPC-based therapy resides in the massive loss of transplanted cells upon delivery, which is a yet-to-be-resolved issue that significantly compromises the therapeutic benefits. The poor retention may be attributed to various intrinsic and extrinsic factors such as pro-apoptotic stressors, inflammation, hypoxia, and oxidative stress (Salazar-Noratto et al., 2020). Genetic activation of FOXO3 has been demonstrated to increase the resistance of MPCs to oxidative stress-induced apoptosis (Yan et al., 2019). Here, we explored whether constitutive activation of FOXO3 in grafted MPCs may be beneficial for counteracting the hostile microenvironment in an ischemic context and found that FOXO3-GE-MPCs were retained longer than WT-MPCs, along with improved therapeutic effects in ischemic heart. Likewise, upregulation and activation of FOXO3 have been reported in renal tubular cells under hypoxic conditions to protect kidney from ischemic injury (Li et al., 2019). Mechanistically, growing evidence indicates that the protective role of FOXO3 against tissue damage may be associated with the expression of antioxidant enzymes and the activation of other pro-survival pathways (Lim et al., 2017; Yan et al., 2019). Therefore, FOXO3-GE-MPCs may provide a feasible option for more effective preclinical and clinical applications targeting ischemic diseases.

Notably, vascular reconstruction has been identified as a key event for the repair of ischemic heart and we have reported downregulation of FOXO3 as a key driver for primate vascular endothelial aging and activation of FOXO3 conferring vascular protection (Yan et al., 2019; Zhang et al., 2020). In this study, we showed that FOXO3-GE-MPCs promoted angiogenesis in the ischemic border zone at least partially via the secretion of pro-angiogenic factors, as evidenced by the higher expression levels of *ESM1*, *FGF16* and *IGF2* in FOXO3-GE-MPCs compared with those in WT-MPCs. Likewise, enhanced migration, clonal expansion and tube formation capacities of HAECs were observed upon the incubation with conditioned medium from FOXO3-GE-MPCs when compared with those in conditioned medium from WT-MPCs, further supporting the notion of possible paracrine effects by FOXO3-GE-MPCs in promoting angiogenesis and cardiac repair against ischemic injury.

In conclusion, genetic activation of FOXO3 extends the retention time of hESC-derived MPCs upon transplantation and confers better therapeutic effects in a mouse model of myocardial infarction. This new finding, together with the previous observations that FOXO3-enhanced hMPCs are resistant to oncogenic transformation (Yan et al., 2019), may support for the safety and effectiveness of using these cells to treat human myocardial infarction diseases in the future.

## Electronic supplementary material

Below is the link to the electronic supplementary material.

Supplementary material 1 (PDF 3006 kb)

Supplementary material 2 (XLSX 11 kb)

Supplementary material 3 (XLSX 11 kb)

Supplementary material 4 (XLSX 120 kb)

Supplementary material 5 (XLSX 377 kb)
