# Supplementary material for: FOXO3-engineered human mesenchymal progenitor cells efficiently promote cardiac repair after myocardial infarction
Source: Protein Cell. 2020 Aug 18;12(2):145–51. doi: 10.1007/s13238-020-00779-7 (PMC7862469; doi:10.1007/s13238-020-00779-7)
Supplement: Supplementary file 1 — Supplementary material 1 (PDF 3006 kb) [file 13238_2020_779_MOESM1_ESM.pdf]

MATERIALS AND METHODS

Animals

Healthy male NOD-SCID mice aged 10-12 weeks were purchased from Beijing Vital River Laboratory Animal Technology Co., Ltd, and were housed in controlled 12-hour light/dark conditions with the intake of water and food *ad libitum*. All animal experiments were performed according to the institutional animal ethics guidelines on the use of laboratory animals and were approved by the Institute of Zoology of the Chinese Academy of Sciences.

Establishment of mouse myocardial infarction model and cell transplantation

Myocardial infarction was induced using a permanent coronary artery occlusion without ventilation method as previously described (Gao et al., 2010). hMPCs were cultured on gelatin-coated plates in MEMα medium (GIBCO) supplemented with 10% FBS, 1% NEAA, 1% penicillin/streptomycin and 1 ng/mL FGF-2. For cell implantation, 2×10^5 WT-MPCs or FOXO3-GE-MPCs suspended in 30 μL MEMα medium were injected at 3 points in the infarct border zone using a 30-gauge syringe immediately after the ligation. 30 μL MEMα medium was injected intramyocardially as vehicle control (Liu et al., 2014).

Assessment of cardiac function

Transthoracic echocardiography was performed to evaluate mouse cardiac function at 4 weeks after myocardial infarction and cell injection using a VisualSonics Vevo 2100 imaging system (Visual Sonics, Inc.) with a 30-MHz transducer. Mice were anesthetized with 2% isoflurane in 100% O_2 gas. Two-dimensional targeted M-mode traces were obtained at the level of the papillary muscle. Left ventricular ejection fraction (EF), fractional shortening (FS), end-diastolic diameter (LVID; d), end-systolic diameter (LVID; s) were measured and calculated using the cardiac echocardiography software from three separate cardiac cycles.

In vivo tracing of transplanted MPCs in mouse heart

Mesenchymal progenitor cells were transfected by lentiviral luciferase before being implanted into the ischemic heart. On days 1, 3, 5, 7, 9 and 11 after the transplantation, mice were injected intraperitoneally with 100 μL of D-luciferin (15 mg/mL), anaesthetized by isoflurane and photographed by an IVIS Lumina XRMS Series III instrument. The region of interest (ROI) of luciferase-labelled MPCs was measured and the data are presented as a relative value to those of WT-MPCs at day 1.

Exercise exhaustion test

Mice were trained to acclimatize to treadmill exercise before an exercise exhaustion test was performed to evaluate their cardiac function. Briefly, mice were placed on treadmill on a 30° incline and started at a warm-up speed of 5 m/min for 4 min. The speed was then set to 14 m/min for 2 min, followed by a 2 m/min increase until exhaustion, which was defined by a mouse being unable to resume running within 10 sec of continuous electric stimulation. The running distance was recorded and analyzed for each mouse.

Heart sampling

At 4 weeks after coronary artery ligation and cell injection, mice were anesthetized by intraperitoneal injection of pentobarbital (50 mg/kg). After the perfusion with saline, the heart was harvested and weighed. For each group, 5 hearts were collected and

divided into left and right ventricles, snap-frozen in liquid nitrogen and stored at -80°C until use. 5 additional hearts for each group were fixed in 4% polyformaldehyde overnight and dehydrated in 30% sucrose, after which transversal frozen sections (10 μm) were prepared for histological assessment.

Immunofluorescence staining

Frozen sections were washed twice with PBS, permeabilized in 0.4% Triton X-100 for 10 min and blocked in 5% BSA in PBS for 30 min at room temperature. The sections were then incubated with primary antibodies diluted in blocking buffer (5% BSA in PBS) overnight at 4°C, followed by the incubation with fluorescent secondary antibodies and nuclear counterstain with Hoechst 33258. Immunofluorescence images were captured by laser scanning confocal microscopy and quantified using ImageJ. Antibodies used in this study are listed in Table S1.

TUNEL staining

Terminal deoxynucleotidyl transferase dUTP nick end labeling (TUNEL) staining was performed to evaluate cardiomyocyte apoptosis by using a one-step TUNEL apoptosis assay kit (C1088, Beyotime). The sections were subsequently stained with anti-cardiac troponin T antibody and Hoechst 33258. The images were captured by laser scanning confocal microscopy and the apoptotic rate of cardiomyocytes was quantified using ImageJ.

Masson's trichrome staining

After washing with PBS, myocardial sections were stained according to the protocol of Masson's Trichrome stain kit (G1340, Solarbio). All images were captured using a digital pathology slide scanner (Aperio CS2, Leica). Infarcted scar size in each heart was calculated as the ratio of total scar area to left ventricular area.

SA-β-gal staining

SA-β-gal staining of hMPCs was performed as previously described (Bi et al., 2020).

***In vitro* tube formation assay**

HAECs were plated at a density of 6×10^3 cells per well onto Matrigel-coated 96-well plates and incubated at 37°C. Cell images were taken after 8 hours of incubation and the cumulative tube length was measured by ImageJ.

***In vitro* wound scratch assay**

The HAECs were plated at a density of 1.5×10^4 cells per well onto collagen-coated 96-well plates and incubated at 37°C until 100% confluence was reached. Cells were scratched once with a wound maker and the medium was changed. Subsequently, the wounding area was photographed once every hour for 16 hours using IncuCyte® Live-Cell analysis system (Essen BioScience, Hertfordshire, UK). The cell-free area was calculated using ImageJ. Migration ability was determined as the percentage of confluence relative to the initial size.

***In vitro* clonal expansion assay**

Clonal expansion assay was performed as previously described with some modifications (Cheng et al., 2019). Briefly, HAECs were seeded at a density of 3×10^3 cells per well in collagen-coated 12-well plates and randomly divided into three experimental groups, FM group (EGM2 medium combined with fresh MPC medium

at a ratio of 1:1), WT-MPC CM group (EGM2 medium combined with WT-MPC conditioned medium at a ratio of 1:1) and FOXO3-GE-MPC CM group (EGM2 medium combined with FOXO3-GE-MPC conditioned medium at a ratio of 1:1). Relative crystal violet-stained area was measured by ImageJ.

Reverse transcription PCR (RT)-PCR and quantitative reverse transcription PCR (RT-qPCR)

For RT-PCR, total RNA was extracted using TRIzol Reagent from mouse hearts. 500 ng of total RNA was reverse-transcribed to cDNA by using the GoScript Reverse Transcription System and oligo (dT) primer. PCR to detect human *GAPDH* in the mouse heart was carried out using Taq DNA Polymerase. Mouse *Gapdh* was used as an internal control. For RT-qPCR, total RNA was extracted using TRIzol Reagent from cultured MPCs. 2 μg of total RNA was reverse transcribed to cDNA as a template for real-time quantitative PCR analysis subsequently performed using a CFX384 Real-Time PCR system with iTaq Universal SYBR Green Super mix. *GAPDH* was used as an internal control. Primers used in this study are listed in Table S2.

Measurement of serum inflammatory factors

Blood samples were collected through the abdominal aorta after the mice were anesthetized using a pro-coagulation tube. After centrifugation at 1,000 *g* for 10 min, serum was collected and used for measurement of inflammatory factors IFN-γ, IL-1β, and TNF-α via radioimmunoassay in collaboration with Beijing North Institute of Biotechnology Co., Ltd.

RNA-seq library construction and sequencing

Total RNA was extracted from the heart tissues from the infarct border zone after MI using the TRIzol reagent according to the manufacturer's protocol. Library construction and sequencing were carried out as previously described (Hu et al., 2020) mixed for each group and divided into 3 technical replicates. 2 μg of total RNA was used to construct sequencing libraries for Illumina by using a NEBNext® Ultra™ RNA Library Prep Kit. The libraries were sequenced on an Illumina HiSeq X-Ten platform.

RNA-seq data analysis

For human MPCs, reads from the previous data (accession number: GSE116277) were aligned to the human genome reference (hg19) with the hisat2 (version 2.0.4) and counted by HTSeq (version 0.11.0). Differentially expressed genes (DEGs) were calculated using R package DESeq2 (version 1.22.2) with a threshold of Benjamini-Hochberg adjusted P value < 0.05 and $|\log_2(\text{fold change})| > 1$. The DEGs were listed in Table S3.

For mouse hearts, low-quality reads were trimmed by TrimGalore (version 0.4.5) and clean reads were mapped to mouse mm10 genome using hisat2 and counted by HTSeq. DEGs were calculated using DESeq2 with a threshold of Benjamini-Hochberg adjusted P value < 0.05 and $|\log_2(\text{Fold change})| > 0.58$ and listed in Table S4. FPKM (fragments per kilobase per million) for each gene was calculated by StringTie (version 1.2.3). Gene ontology (GO) analysis (Biological Process) was conducted by using ToppGene (https://toppgene.cchmc.org/) by default parameters.

Statistical analysis
Two-tailed Student's *t* test or one-way ANOVA followed by Dunnett's test was performed as appropriate by GraphPad Prism 8.0 to assess the difference between groups. Data are presented as the mean ± SEMs. HAECs wound scratch assay was analyzed by two-way ANOVA followed by Dunnett's test. $P < 0.05$ is considered statistically significant.

Data Availability
RNA-seq data have been deposited in the NCBI Gene Expression Omnibus (GEO) under the accession numbers GSE149594 for mouse heart tissues and GSE116277 for human MPCs.

Figure S1. Characterization of FOXO3-GE-MPCs *in vitro* and RNA sequencing analysis of tissues in the infarct border zone of mouse hearts transplanted with FOXO3-GE-MPCs after myocardial infarction.

(**A**) Fluorescence-activated cell sorting (FACS) analysis of MPC-specific markers (CD105, CD90, and CD73) and MPC-irrelevant markers (CD45, CD43 and CD34) in WT-MPCs and FOXO3-GE-MPCs.

(**B**) Cell morphology of WT-MPCs and FOXO3-GE-MPCs (left) and DNA sequencing analysis (right) showing the nucleotide substitutions in FOXO3-GE-MPC genomic DNA that resulted in the replacement of two serines at 253 and 315 with alanines in the FOXO3 protein. Scale bar, 100 μm. Red denotes the original amino acid or nucleotide in the WT-MPCs; blue denotes the edited ones in FOXO3-GE-MPCs.

(**C**) Ki67 immunofluorescence staining in WT-MPCs and FOXO3-GE-MPCs (P7). Scale bar, 25 μm. Quantitative data are shown as the mean ± SEMs. n = 3. **, $P < 0.01$ (two-tailed *t* test).

(**D**) Analysis of clonal expansion ability in WT-MPCs and FOXO3-GE-MPCs (P7). Left, representative images of crystal violet staining. Right, the relative area of crystal violet-positive cells calculated by ImageJ and shown as the mean ± SEMs, n = 3. **, $P < 0.01$ (two-tailed *t* test).

(**E**) SA-β-gal staining of WT-MPCs and FOXO3-GE-MPCs (P7). Scale bar, 50 μm. Data are shown as the mean ± SEMs, n = 3. **, $P < 0.01$ (two-tailed *t* test).

(**F**) Evaluation of apoptotic cardiomyocytes in mouse hearts immunostained with antibodies against α-actinin (green) and cleaved-caspase 3 (red). Scale bar, 25 μm. Quantitative data are shown as the mean ± SEMs. n = 3 for sham group; n = 5 for the other groups. ***, $P < 0.001$; ns, not significant (one-way ANOVA followed by Dunnett's test).

(**G**) Principal component analysis (PCA) showing the gene expression patterns of heart tissues from the infarct border zone with the indicated treatments. Each point represents a sample. Data points were computed based on $\log_2$ (FPKM + 1).

(**H**) Heatmap showing the pairwise Euclidian distance between the indicated samples.

(**I**) Venn diagram showing the numbers of overlapping genes that were upregulated upon MI and downregulated upon the transplantation of FOXO3-GE-MPCs.

(**J**) Volcano plots showing differentially expressed genes (DEGs) between the indicated groups. DEGs were identified by a threshold of $|\log_2$ (fold change)$| > 0.58$ and adjusted *P* value (padj) < 0.05.

(**K**) Representative immunofluorescence images of RelA-positive cells in the infarct

border zone at 4 weeks after MI, Scale bar, 25 μm. The white dotted line denotes the lesion border of the heart. BZ, border zone; IZ, infarct zone. Zoomed-in areas in white rectangles are shown in the lower panel.

(**L**) Tree plot showing the regulatory network of NF-κB and its target genes. Pink rings denote the overlapping genes identified in panel I.

Figure S2. Immunostaining and RT-qPCR analysis of pro-inflammatory factors and vascular markers in the infarct border zone of mouse hearts transplanted with FOXO3-GE-MPCs.

(**A**) RT-qPCR analysis of the relative expression levels of the NF-κB target genes that were indicated to be rescued by FOXO3-GE-MPC transplantation according to our RNA-seq data. n = 3 for sham group; n = 5 for the other groups. *, $P < 0.05$; **, $P < 0.01$; ***, $P < 0.001$ (one-way ANOVA followed by Dunnett's test).

(**B**) Representative immunofluorescence images of ORF1p-positive cells in the infarct border zone at 4 weeks after MI, Scale bar, 25 μm. The white dotted line denotes the lesion border of the heart. BZ, border zone; IZ, infarct zone. Zoomed-in areas in white rectangles are shown in the lower panel.

(**C**) Representative immunofluorescence images of the endothelial cell marker CD31 in the infarct border zone at 4 weeks post MI. Scale bar, 25 μm. The white dotted line denotes the lesion border of the heart. BZ, border zone; IZ, infarct zone. Zoomed-in areas in white rectangles are shown in the lower panel.

(**D**) Representative immunofluorescence images of the vascular smooth muscle cell marker α-SMA in the infarct border zone at 4 weeks post MI. Scale bar, 25 μm. The white dotted line denotes the lesion border of the heart. BZ, border zone; IZ, infarct zone. Zoomed-in areas in white rectangles are shown in the lower panel.

(**E**) Representative images of crystal violet staining for clonal expansion assay.

(**F**) Representative images of wound scratch assay. Images were taken at the beginning (0 h) of the experiment and 13 hours (13 h) after the scratch was performed. Black line denotes the wound edge. Scale bar, 200 μm.

(**G**) Representative images of tube formation assay. Scale bar, 200 μm.

Supplementary Table Legends

Table S1. Antibodies used in this study.

Table S2. Primers used for RT-PCR and RT-qPCR.

Table S3. DEGs identified in FOXO3-GE-MPCs and WT-MPCs, related to Figure 2.

Table S4. DEGs identified in infarcted mouse hearts transplanted with FOXO3-GE-MPCs or vehicle and infarcted mouse hearts with MI or sham, related to Figure 2 and Figure S1.

REFERENCES

Bi, S., Liu, Z., Wu, Z., Wang, Z., Liu, X., Wang, S., Ren, J., Yao, Y., Zhang, W., Song, M.*, et al.* (2020). SIRT7 antagonizes human stem cell aging as a heterochromatin stabilizer. Protein Cell 11, 483-504.

Cheng, F., Wang, S., Song, M., Liu, Z., Liu, P., Wang, L., Wang, Y., Zhao, Q., Yan, K., Chan, P.*, et al.* (2019). DJ-1 is dispensable for human stem cell homeostasis. Protein Cell 10, 846-853.

Gao, E., Lei, Y.H., Shang, X., Huang, Z.M., Zuo, L., Boucher, M., Fan, Q., Chuprun, J.K., Ma, X.L., and Koch, W.J. (2010). A novel and efficient model of coronary artery ligation and myocardial infarction in the mouse. Circ Res 107, 1445-1453.

Hu, H., Ji, Q., Song, M., Ren, J., Liu, Z., Wang, Z., Liu, X., Yan, K., Hu, J., Jing, Y.*, et al.* (2020).

ZKSCAN3 counteracts cellular senescence by stabilizing heterochromatin. Nucleic Acids Res 48, 6001-6018.
Liu, X., Chen, H., Zhu, W., Hu, X., Jiang, Z., Xu, Y., Zhou, Y., Wang, K., Wang, L., Chen, P., *et al.* (2014). Transplantation of SIRT1-engineered aged mesenchymal stem cells improves cardiac function in a rat myocardial infarction model. J Heart Lung Transplant 33, 1083-1092.

Figure S1

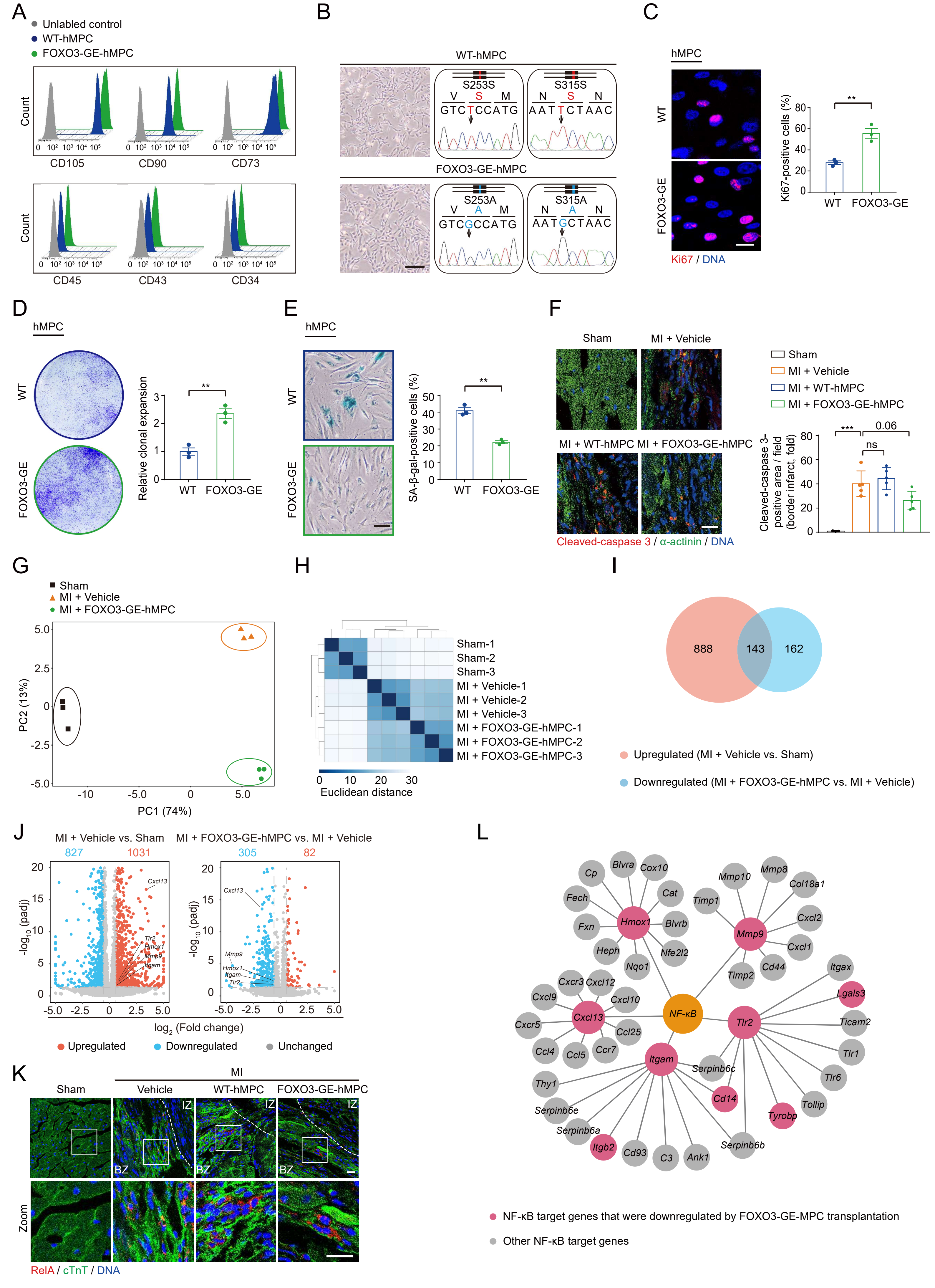
A
Unlabled control
WT-hMPC
FOXO3-GE-hMPC
Count
CD105
CD90
CD73
CD45
CD43
CD34
B
WT-hMPC
S253S
S315S
FOXO3-GE-hMPC
S253A
S315A
C
hMPC
WT
FOXO3-GE
Ki67 / DNA
Ki67-positive cells (%)
D
hMPC
Relative clonal expansion
E
SA-β-gal-positive cells (%)
F
Sham
MI + Vehicle
MI + WT-hMPC
MI + FOXO3-GE-hMPC
Cleaved-caspase 3 / α-actinin / DNA
Cleaved-caspase 3-positive area / field (border infarct, fold)
G
PC1 (74%)
PC2 (13%)
H
Sham-1
Sham-2
Sham-3
MI + Vehicle-1
MI + Vehicle-2
MI + Vehicle-3
MI + FOXO3-GE-hMPC-1
MI + FOXO3-GE-hMPC-2
MI + FOXO3-GE-hMPC-3
Euclidean distance
I
888
143
162
Upregulated (MI + Vehicle vs. Sham)
Downregulated (MI + FOXO3-GE-hMPC vs. MI + Vehicle)
J
MI + Vehicle vs. Sham
827
1031
MI + FOXO3-GE-hMPC vs. MI + Vehicle
305
82
-log10 (padj)
log2 (Fold change)
Upregulated
Downregulated
Unchanged
K
MI
Sham
Vehicle
WT-hMPC
FOXO3-GE-hMPC
IZ
BZ
Zoom
RelA / cTnT / DNA
L
NF-κB
Hmox1
Mmp9
Cxcl13
Tlr2
Itgam
NF-κB target genes that were downregulated by FOXO3-GE-MPC transplantation
Other NF-κB target genes

Figure S2

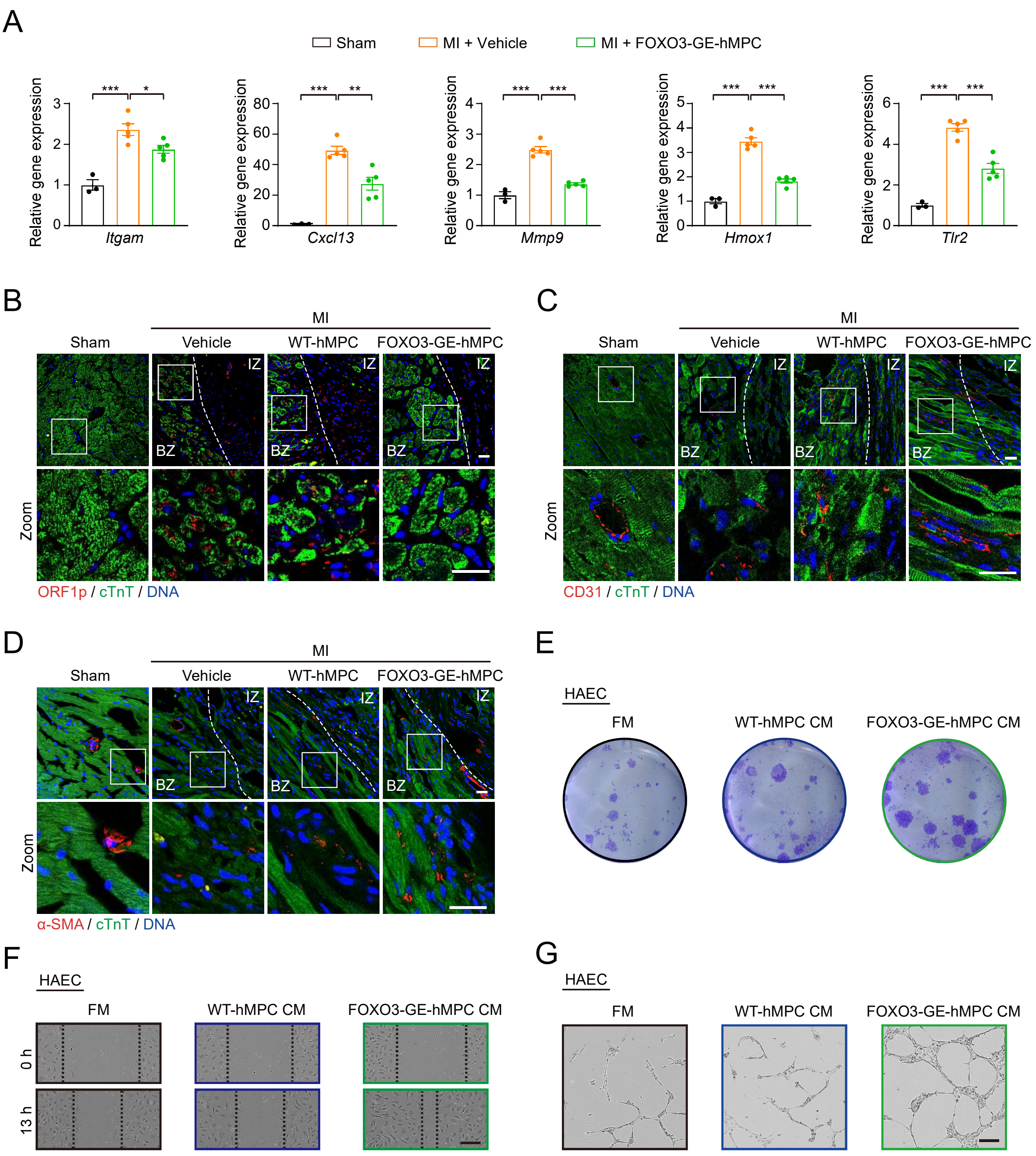
A
Sham
MI + Vehicle
MI + FOXO3-GE-hMPC
Relative gene expression
Itgam
Cxcl13
Mmp9
Hmox1
Tlr2
B
MI
Sham
Vehicle
WT-hMPC
FOXO3-GE-hMPC
IZ
BZ
Zoom
ORF1p / cTnT / DNA
C
CD31 / cTnT / DNA
D
α-SMA / cTnT / DNA
E
HAEC
FM
WT-hMPC CM
FOXO3-GE-hMPC CM
F
0 h
13 h
G
